# A Rubric to Assess the Design and Intervention Quality of Randomized Controlled Trials in Health and Wellness Coaching

**DOI:** 10.1177/15598276221117089

**Published:** 2022-08-08

**Authors:** Sebastian Harenberg, Gary A. Sforzo, Joel Edman

**Affiliations:** Department of Human Kinetics, 1270 St Francis Xavier University, Antigonish, NS, Canada (SH); Exercise & Sport Sciences, 4102 Ithaca College, Ithaca, NY, USA (GAS); and Edman Wellness Services, Media, PA, USA (JE)

**Keywords:** Health coaching, wellness coaching, obesity, type 2 diabetes, behavior change, dietary guidelines, exercise

## Abstract

**Objective:**

To collect health and wellness coaching (HWC) literature related to treatment of obesity and Type 2 Diabetes (T2D) for systematic assessment using a novel rubric.

**Data Source:**

Pubmed, CINAHL, and PsychInfo

**Study Inclusion and Exclusion:**

Given 282 articles retrieved, only randomized and controlled trials meeting a HWC criteria-based definition were included; studies with intervention <4 months or <4 sessions were excluded.

**Data Extraction:**

Rubric assessment required details of two theoretical frameworks (i.e., study design and HWC intervention design) be extracted from each included paper.

**Data Synthesis:**

Data were derived from a 28-item rubric querying items such as sampling characteristics, statistical methods, coach characteristics, HWC strategy, and intervention fidelity.

**Results:**

29 articles were reviewed. Inter-rater rubric scoring yielded high intraclass correlation (r = .85). Rubric assessment of HWC literature resulted in moderate scores (56.7%), with study design scoring higher than intervention design; within intervention design, T2D studies scored higher than obesity.

**Conclusions:**

A novel research design rubric is presented and successfully applied to assess HWC research related to treatment of obesity and T2D. Most studies reported beneficial clinical findings; however, rubric results revealed moderate scores for study and intervention design. Implications for future HWC research are discussed.

“Rather than simply making general comments, we sought to develop a tool to bring consistency and objectivity to assessing HWC.”

Health and wellness coaching (HWC) is an intervention strategy, promoting healthy behavioral change and thereby minimizing potential for adverse health outcomes.^1-3^ More specifically, HWC is a patient-centric process supporting individualized goals often related to eating well, exercising regularly, managing stress effectively, and identifying important resources to promote healthful living.^4^ An extensive and growing evidence-base for HWC exists describing prospects for both treating and preventing these disorders.^5,6^ However, methodological questions related to HWC research exist. While a systematic review has shed some light on the definition of HWC,^7^ the HWC strategies applied during intervention are of particular concern in the present study.^8^ A systematic and effective assessment in this area of extant HWC literature is lacking.

There are over 100 randomized controlled trials (RCTs) of HWC interventions.^5,6^ RCTs are widely considered the gold standard of original research required for advancing medical knowledge. However, RCTs can contain design shortcomings (e.g., uncontrolled concomitant treatments, underpowered, selection bias) impacting study quality and the unbiased results that are sought.^9,10^ There are many evaluations of the quality of RCTs (e.g., psychological interventions by Temple et al.^11^). Such evaluations of research allow policymakers, and clinicians, to make informed decisions about implementing psychological treatments in clinical services. Though assessment of quality for psychological interventions exists,^9-11^ such inspection of HWC research does not. As HWC moves toward becoming an important and credible health and medical intervention, it seems reasonable and potentially valuable to develop a systematic means to assess relevant RCTs.

The purpose of this review is to evaluate the quality of RCTs in HWC research. Because a review of all available RCTs would not be feasible, this work will focus on some of the most pressing present health crises, obesity and T2D.^12,13^ Obesity increases the risk of various cardiovascular diseases and other comorbid symptoms and diseases, while T2D is a chronic disease leading to life-threatening health consequences (e.g., atherosclerosis, renal failure, blindness).^14,15^ Moreover, these disorders are linked, with obesity and subsequent insulin resistance leading to the development of T2D.^15^ Effective prevention and management of these conditions is necessary for optimization of well-functioning and successful contemporary healthcare systems. Therefore, it is essential to have credible research describing effective interventions for obesity and T2D. Such evidence may enable future HWC researchers and practitioners to learn from best practices in HWC research and provide valuable information for evidence-based practice.

To achieve the purpose of the present study, a comprehensive scoring system for HWC research was developed and applied. The HWC Research Design Rubric (HWC-RDR) with supportive criteria is introduced, and the evaluative review of obesity and T2D literature is presented. We chose obesity and T2D because there is a critical mass of research with these conditions being the most frequently studied in HWC literature.^5,6^ Data from the HWC-RDR are used to illustrate the strengths of, and challenges to, HWC literature.

## Methods

### Overview

This project was completed in three phases. First, a systematic review of the health coaching literature produced RCTs describing HWC as an intervention for obesity and T2D. Second, a research scoring rubric was developed and then selected RCTs were assessed (for study design and HWC intervention design) using the rubric. Finally, rubric-generated data were inspected and analyzed to show trends and allow discussion of HWC research. Greater detail on these three methodological phases is provided below.

### Literature Search Strategy

**Figure 1.**
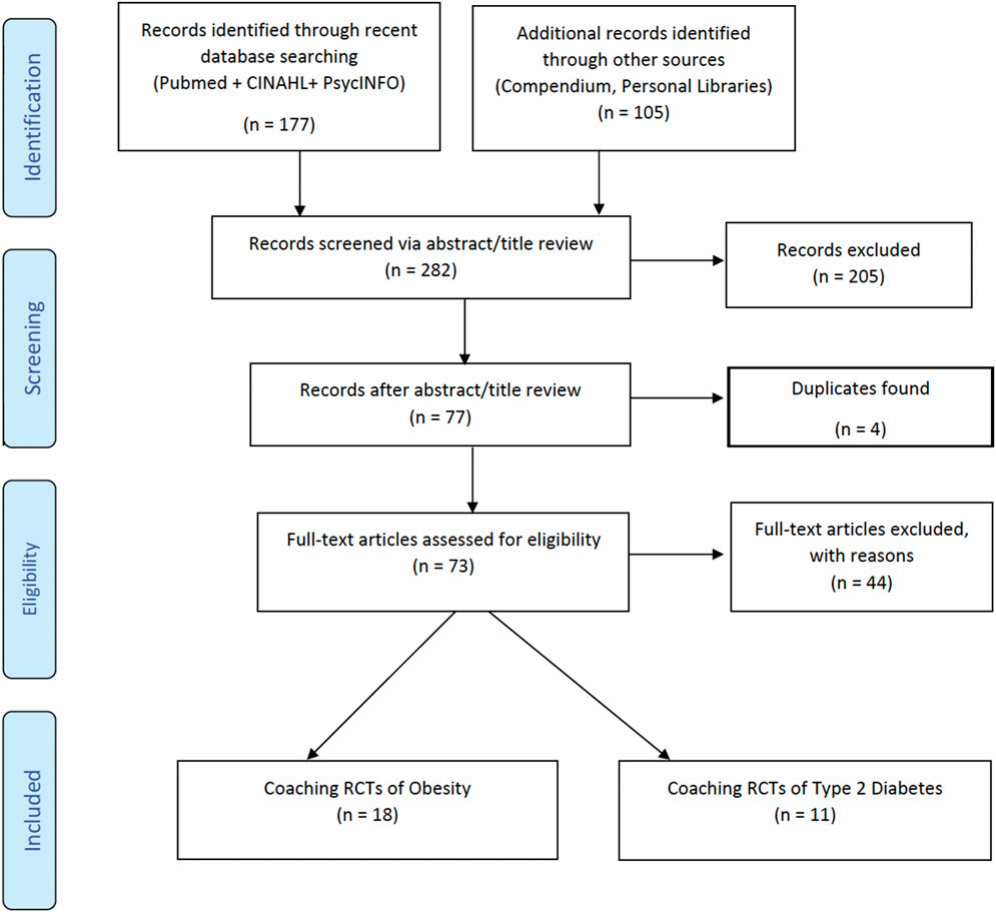
PRISMA diagram showing article selection process.

A two-part strategy was used to locate relevant HWC literature. First, all eligible articles in the Compendium of HWC Literature related to obesity and T2D were identified.^5,6^ In total, 41 randomized studies in obesity and T2D were part of the original compendium. This set of studies was complemented by articles from personal libraries, for a total of 105. Second, a professional librarian using search strategies previously applied for the Compendium review,^5^ identifying recent articles (i.e., published after the compendium) to add to that literature. In the search, 177 additional abstracts were identified. All papers retrieved from the compendium and the new search were filtered using HWC inclusion criteria as applied previously.^5^ After the review of abstracts and removal of duplicates, 209 records were excluded. The remaining 73 articles were screened for eligibility. Inclusion criteria were as follows: The study had to be an RCT and the intervention of the RCTs had to have at least four coaching sessions over a four-month period allowing ample time for behavior change and providing an opportunity for coach-patient relationship development.^4^ As seen in the accompanying PRISMA flow chart (Figure 1), ultimately, 29 RCTs (18 obesity and 11 T2D) were included for review.

### Development of HWC Research Design Rubric

In general, the rubric was developed using previously established research quality assessment tools and input from HWC subject-matter experts. The HWC-RDR was designed for use with all HWC research and questions are not specific to obesity and T2D. There are two theoretical frameworks shaping the HWC-RDR: (A) Study Design, and (B) Intervention Design with each section having three subcategories.

#### A. Study Design Rubric

The study design framework was developed by reviewing recent articles on the evaluation of RCTs in psychological disorders (i.e., emotional distress in breast cancer;^11^ eating disorder prevention;^9^ depression and neurosis.^10^ The aim was to generate a brief set of questions covering the most important aspects of HWC study design. The main conceptual areas included (1) the acquisition of participants (i.e., recruitment type, clear inclusion and exclusion criteria, reporting sample characteristics, sample size determination), (2) RCT design (i.e., allocation, concealment, blinding, control of confounding treatment, outcome measure quality), and (3) statistical analyses (i.e., baseline comparison, intent-to-treat analysis, appropriate analysis, control for covariates). The study design section included 15 questions—scoring range 0–1 for 7 questions and 0–2 for 8 questions, with a total maximum design score of 23 (see Table 1).

**Table 1.** Health and Wellness Coaching Research Design Rubric: Study Design Criteria.

1.Participant Recruitment	2.Random Allocation	3.Allocation Concealment	4.Assessor Blinding	5.Exclusion/Inclusion Criteria	6.#Excluded/Withdrawn Reported
0 = Convenience 1 = Random	0 = Non-Random 1 = Cluster Random 2 = True Random	0 = not reported or not concealed 1 = concealed	0 = not reported or aware 1 = unaware	0 = no or not reported 1 = Yes	0 = no or not reported 1 = Yes
7.Sample Characteristics	8.Sample Size Determination	9.Active Controls	10.Baseline	11.Intent-to-treat	12.Quality of Outcome
0 = no or not reported 1 = Yes	0 = not reported 1 = mentioned 2 = power analysis	0 = not reported, no, or usual care 1 = Yes, low fidelity 2 = Yes, high fidelity/sham	0 = not checked 1 = not equivalent 2 = equivalent	0 = no or not reported 1 = Yes	0 = not validated 1 = established but weak details 2 = Validated
13.Appropriateness of Statistics	14.Control of Covariates	15. Confounding Treatment			
0 = not clear 1 = not fully justified 2 = well justified	0 = no or not reported 2 = Yes	0 = present 1 = standardized across groups 2 = no confounders			

Abbreviations: HWC, Health and Wellness Coaching.

#### B. Intervention Design Rubric

Most items for the HWC intervention design framework were derived from the job task analysis of HWC.^4^ Input and discussion from HWC subject-matter experts were also sought and incorporated into item formation. The intervention design questions represented three main conceptual areas: (1) Coach qualities (i.e., education, training, and experience); (2) HWC program definition and design (i.e., client centered, goal setting, accountability, personal relationship, session length and number, and program duration); and (3) Adherence to HWC strategy (i.e., using established behavior change principles and ensuring fidelity of HWC application). The intervention design section included 13 questions with 0–2 response scores for a maximum score of 26 (see Table 2).

**Table 2.** Health and Wellness Coaching Research Design Rubric: Intervention Design Criteria.

1.Coach Training	2.Coach Certification	3.Coach Academic Degree	4.Coach Clinical Experience	5.Coaching Experience	6.Personal Delivery	7.Coaching Frequency
0 = not reported 1 = Trained but not NBHWC 2 = NBHWC training or equivalent	0 = not reported 1 = certified but not NBHWC equivalent 2 = NBHWC or equivalent	0 = not reported or none 1 = A.S. in healthcare 2 = B.S. in healthcare	0 = not reported or none 1 = 2 y in healthcare clinic 2 = >2 y in healthcare clinic	0 = not reported or none 1 = <1 y 2 = >1 y	0 = not reported 1 = group or >1 coach 2 = 1 coach	0 = not reported or <1/mo 1 = 1/mo for 3–6 mo 2 = 2x/mo for >3 mo

8. Duration of Sessions	9.Duration of Program	10. Client centered	11.Coaching Process Defined	12. Client Coaching Adherence	13.Coaching Supervision	
0 = not reported or <15 min 1 = 15-25 min 2 = >25 min	0 = not reported or <3mo 1 = 3mo but <6 mo 2 = >6 mo	0 = not reported or not done 1 = Health coach designated goals 2 = client directed goals	0 = not reported 1 = partially with behavior change theory 2 = comprehensive description of behavior change program	0 = not reported or <50% 1 = 50-75% 2 = >75%	0 = not reported or done 1 = team meeting 1/mo 2 = mentorship regularly	

Abbreviations: HWC, Health and Wellness Coaching; NB, National Board.

### Reviewing Process and Reliability Check

All studies were reviewed by all authors and general scoring system questions clarified before applying the HWC Research Design Rubric. Subsequently, the first author scored all articles using the study design questions from the rubric. The second author scored all articles using the rubric’s intervention design questions. The third author scored all included articles using all rubric questions. The third author scores were compared to the first two authors’ scores using intraclass correlation coefficients which yielded very good ratings of agreement (ICC = .85; CI95% = .68-.93).

### Data Summary and Statistical Analysis

After scoring all included articles with the HWC-RDR, variables represented with continuous data were summarized as means, medians, and standard deviations. All categorical data were expressed in counts and percentages. Before parametric statistics were performed, normality and appropriate assumptions for each test were checked. For comparison of continuous data between obesity and diabetes studies, independent t-test with Cohen’s d effect sizes were calculated. Relationships between variables were assessed via Pearson’s correlation coefficients. Alpha was set at .05 and statistical analyses were conducted using JASP .14.1 or SPSS 27. To assist discussion of best research practices, the top three rubric scoring obesity and T2D papers were identified and used to illustrate study and intervention design highlights.

## Results

### Descriptive

There were 29 peer-reviewed RCTs selected for review and HWC-RDR scoring, including 18 obesity and 11 T2D studies. Table 3 provides methodological highlights and outcome summary for all included articles.^16-44^ The studies’ average HWC intervention time was 11.55 months (SD = 6.64, range = 3–24, median = 12). The average study sample size was 338.48 participants (SD = 390.26, range = 25–1755, median = 190). Only one included study (3.4%) was published before 2010. More than half of the studies (N = 16, 55.1%) were conducted between 2015 and 2018 (see Table 3).

**Table 3.** Overview of Included Studies.

Diabetes	Population Studied	Sample (N)	Length (mo)	HC Sessions	Coaching Delivery	A1c	Weight/BMI
Chapman et al. (2018)	Community health center adults	588	18	41	Both	(+)	
Cinar et al. (2018)	Adults, 30+ yrs old	302	12	5–7	Both	(+)	
Fischer et al. (2012)	Low-income adults	762	20		Telephonic	0	
Kempf et al. (2017)	Overweight or obese adults	202	12	12	Telephonic	(+)	(+)
Nishita et al. (2012)	Adults, 25+ yrs old, on 2+ DM meds	190	12	14	In-person	0	(+)
Patja et al. (2012)	Adults, 45+ yrs old	1129	12	11	Telephonic	0	
Walker et al. (2011)	Urban adults	526	12	Up to 10	Telephonic	(+)	
Wayne et al. (2015)	Lower socioeconomic status adults	131	6		Telephonic (and smart phone)	(+)	(+)
Willard–Grace et al. (2015)	Low-income adults	441	12	12.4	Both	(+)	
Wolever et al. (2010)	Adults on DM medication, 1+ yrs	52	6	14	Telephonic	(+)	
Young et al. (2014)	Rural community adults	101	9	5	Telephonic		

Obesity	Description	Sample N	Length (mo)	HC Sessions	Coaching Delivery	Weight/BMI	
Alencar et al. (2017)	Obese adults	25	3	12	Video conference call	(+)	
Allman–Farinelli et al. (2016)	Young adults	250	9	7	Telephonic (text messages)	(+)	
Annesi et al. (2016)	Adult obese women	110	24	32	In-person (individual-groups)	(+)	
Appel et al. (2011)	Adults with 1+ CVD risk factors	415	24	33–49	Telephonic arm and In-person arm (individual-groups)	(+)	
Ball et al. (2011)	Obese adolescents	46	5	16	In-person	(+)	
Godino et al. (2016)	Overweight and obese young adults	404	24	Up to 10	Telephonic (social media, text, email)	(+)	
Hersey et al. (2012)	Overweight and obese adults	1755	18	18	Telephonic	(+)	
Huber et al. (2015)	Obese adults from primary care practice	90	6	7	Telephonic	(+)	
Johnson et al. (2018)	Obese adults	30	4	12	Video conference and in-person arms	(+)	
Leahey et al. (2013)	Obese adults, 40–60 yrs old	44	6	12	In-person groups (professional, peer and mentor)	(+)	
Lin et al. (2016)	Middle aged women-metabolic syndrome risk	115	3	12	Telephonic		
McCarthy et al. (2017)	Overweight and obese soldiers	435	3	Up to 3	Telephonic (or email)	0	
Nguyen et al. (2013)	Overweight and obese adolescents	151	24	26	Both (indiviual-groups)	0	
Rimmer et al. (2009)	Obese AA women with mobility disabilities	92	6	30	Telephonic (monthly support groups)	(+)	
Rimmer et al. (2013)	Adults with physical disabilities	102	9	25	Telephonic	(+)	
Simpson et al. (2015)	Obese adults	166	12	15	Both	(+)	
Steinberg et al. (2017)	Overweight and obese AA women	184	12	12	Telephonic		
Taveras et al. (2017)	At risk children	721	12	6	Telephonic or video call (texting)	0	

Abbreviations: HC = Health coaching; BMI = body mass index; A1c = glycosylated hemoglobin; + = beneficial significant finding; 0 = no change; AA = African American.

### HWC Research Design Rubric Scores

Total HWC-RDR score is the sum of study design and intervention design scores from all rubric items. Average total HWC-RDR score across all studies was 27.8/49 (SD = 4.73, range = 16–35, median = 28) or 56.7%. This overall score indicates moderate ranking for this HWC literature base describing intervention for obesity and T2D. The evaluated studies were better at general study design characteristics than planning and describing application of HWC. This is not surprising given that HWC is a relatively new clinical approach and evolving as an allied healthcare field.^45,46^ Strengths and weaknesses for study design and intervention design, are described and discussed below.

The mean overall HWC-RDR score for obesity studies was 26.3 (SD = 4.99) and for diabetes was 30.1 (SD = 3.24). These data showed sufficient normality (i.e., non-significant Shapiro–Wilk tests) and homogeneity of variance (i.e., insignificant Levene’s test) to warrant use of parametric statistics. Obesity studies showed a significantly lower total research design rubric score than diabetes studies (t_(27)_ = 2.22, p = .035, Cohen’s d = .85, mean difference = 3.76, CI95% = .28–7.23). This difference was a function of intervention design scores and is discussed below.

#### A. Study Design Scores

Average study design score for the 29 studies was 15.1/23 (SD = 2.42, range = 9–19, median = 15) or 65.6%. When looked at by condition, the mean study design score for obesity studies was 14.60 (SD = 2.66) and for diabetes was 15.3 (SD = 2.30). These data showed sufficient normality (i.e., non-significant Shapiro–Wilk tests) and homogeneity of variance (i.e., insignificant Levene’s test) to warrant use of parametric statistics. Study design scores did not differ significantly between obesity and diabetes studies (t_(27)_ = .75, p = .46, Cohen’s d = .23) indicating similarity in research design.

Table 4 provides study design scoring for each HWC-RDR question while Table 5 presents rubric scoring raw data by article. For study design, low overall mean scores (<30%) were reported for recruitment, concealment, blinding, and intent-to-treat questions. High overall mean scores (>70%) were reported for allocation, statistics, covariates, and confounders while moderate scores (>30 <70%) were found for the remaining study design rubric questions.

**Table 4.** Health and Wellness Coaching Research Design Rubric Scores by Item.

Rubric Question	Diabetes Data	Obesity Data	Overall
Study Design	(M + SD)	(M = SD)	(M + SD)
1. Recruitment	.09 +.30	.00 +.00	.03 +.19
2. Allocation	1.82 +.041	1.94 +.24	1.90 +.31
3. Concealment	.18 +.41	.22 +.43	.21 +.41
4. Blinding	.18 +.41	.33 +.49	.28 +.46
5. In/Exclusion	1.00 +.00	1.00 +.00	1.00 +.00
6. In/Ex reported	1.00 +.00	.94 +.24	.97 +.19
7. Sample	1.00 +.00	.94 +.24	.97 +.19
8. Power	1.27 +1.01	1.22 +.81	1.24 +.87
9. Controls	.55 +.82	.72 +.58	.66 +.67
10. Baseline	1.27 +1.01	1.22 + .94	1.24 +.95
11. Intent-to-Treat	.73 +.47	.83 +.038	.79 +.41
12. Outcomes	1.00 +.00	1.06 +.24	1.03 +.19
13. Statistics	1.67 +.51	1.83 +.38	1.76 +.44
14. Covariates	1.09 +1.05	1.67 +.77	1.45 +.91
15. Confounders	1.82 +.41	1.39 +.85	1.55 +.74

INTERVENTION DESIGN			
1. Coach training	1.00 +.00	.65 +.49	.79 +.42
2. Certification	.00 +.00	.00 +.00	.00 +.00
3. Education	1.55 +.82	1.29 +.92	1.39 +.86
4. Clinical experience	.91 +.54	.41 +.62	.61 +.63
5. Coaching experience	.27 +.64	.12 +.33	.18 +.48
6. Method of delivery	1.73 +.47	1.47 +.72	1.57 +.63
7. Session frequency	1.36 +.67	1.53 +.72	1.46 +.69
8. Session duration	1.18 +.98	.59 +.71	.82 +.86
9. Program length	2.00 +.00	1.65 +.49	1.79 +.42
10. Client-centered	1.73 +.47	1.24 +.75	1.43 +.69
11. Coaching defined	1.46 +.52	1.06 +.66	1.21 +.63
12. Session adherence	1.27 +.91	.65 +.86	.89 +.92
13. Supervision/Fidelity	1.00 +.78	.53 +.87	.71 +.86

**Table 5.** Health and Wellness Coaching Research Design Rubric Scores by Randomized Controlled Trials.

Diabetes	Design Score (23)	Intervention Score (26)	Total Score (53)
Chapman et al. (2018)	14	14	28
Cinar et al. (2018)	15	17	32
Fischer et al. (2012)	17	12	29
Kempf et al. (2017)	19	12	31
Nishita et al. (2012)	15	19	34
Patja et al. (2012)	15	19	34
Walker et al. (2011)	13	10	24
Wayne et al. (2015)	14	17	31
Willard–Grace et al. (2015)	17	12	29
Wolever et al. (2010)	12	21	33
Young et al. (2014)	9	17	26

OBESITY	Design Score (23)	Intervention Score (26)	Total Score (53)
Alencar et al. (2017)	15	9	24
Allman–Farinelli et al. (2016)	19	8	27
Annesi et al. (2016)	11	8	19
Appel et al. (2011)	19	14	33
Ball et al. (2011)	15	16	31
Godino et al. (2016)	17	6	23
Hersey et al. (2012)	16	10	26
Huber et al. (2015)	13	14	27
Johnson et al. (2018)	11	8	19
Leahey et al. (2013)	16	13	29
Lin et al. (2016)	16	15	31
McCarthy et al. (2017)	13	3	16
Nguyen et al. (2013)	18	8	26
Rimmer et al. (2009)	16	12	28
Rimmer et al. (2013)	15	15	30
Simpson et al. (2015)	16	19	35
Steinberg et al. (2017)	16	8	24
Taveras et al. (2017)	14	12	26

#### B. Intervention Design Scores

Average intervention design scores for the 29 studies was 12.9/26 (SD = 4.30, range = 3–21, median = 13) or 49.6%. Looking by condition, the mean for obesity studies was 11.3 (SD = 4.07) and diabetes studies was 15.5 (SD = 3.62). These data showed sufficient normality (i.e., non-significant Shapiro–Wilk tests) and homogeneity of variance (i.e., insignificant Levene’s test) to warrant use of parametric statistics. Obesity studies showed significantly lower HWC intervention design scores compared to diabetes (t_(27)_ = 2.97, p = .0006, Cohen’s d = 1.14, mean difference = 4.45, CI95% = 1.38–7.53) indicating better HWC intervention design for T2D than obesity studies. Rubric scores were better on nearly every intervention design item in the T2D studies with noticeably greater scores on session duration, adherence, and fidelity questions. It is not clear why intervention design scores were better in the T2D studies. Future HWC research should model the best of these for optimization of interventions design in all HWC studies.

Table 4 provides HWC intervention design rubric scoring for each question while Table 5 provides intervention design raw data and scoring for each study. Examining intervention design questions, method of delivery, frequency, program length, and client-centered rubric questions rated overall high mean scores. Moderate overall mean scores were reported for questions related to coach training, education, clinical experience, coaching definition, session adherence, and intervention fidelity. Low overall mean intervention design scores were found for certification and coaching experience questions.

To allow clinical application or methodological replication, it is necessary to elaborate details of an intervention trial. Specific to HWC, Hill et al.^8^ and Olsen and Nesbit,^47^ called for the need to clearly describe the intervention. Simpson et al^31^ provided an excellent description of intervention components and coaching theory. For many papers, however, a low intervention design score was often related to inadequate description of the HWC coaches and strategy. Regarding coaching background, very few studies described coaching experience, and none had coaches with certification. National board certification (NBC-HWC^48^) was only established in 2017, so it was not surprising none of the included RCTs mentioned this valued but recently recognized national credential. Only 2/18 (11.1%) obesity and 2/11 (18.2%) T2D studies described employing health coaches with ample coaching experience. Wolever et al.^43^ had a very thorough description of coach background and training. This is valuable because using well-trained and experienced coaches seems essential to providing a quality intervention.

### Publication Year, Sample Size, Intervention Length

Total HWC-RDR score was negatively related to publication year (r = −.43, p = .02, CI95% = −.07 to −.69) indicating more recent publications were associated with lower overall HWC Research Design Rubric scores. Non-significant correlations were found between total rubric score and sample size (r = −.36, p = .06) and intervention length (r = .04, p = .85).

It is interesting to note that the best scoring articles from this review were conducted in the late 2000s and early 2010s. A negative correlation between year of publication and rubric score implies a decline in quality of study design in recent publications. One possible explanation is the recent increase in internet publications contributing to quality decline^49^ while pressure to publish may also be lowering research quality.^50^ Alternatively, this may represent a small sample anomaly with a timeline of only 10 years reflected in the analysis.

## Discussion

The purpose of the present study was to evaluate the quality of RCTs focusing on HWC in obesity and T2D. Using the generated HWC-RDR, the evaluation revealed key strengths and areas of improvements for future research. The HWC-RDR is a usable instrument in this context. A key strength is that evaluates the study design element as well as the intervention design, which are both critical to the quality of an RCT in HWC. The usability of the rubric extends past the fields of obesity and T2D and may also be given consideration when designing new RCTs in the field across a range of disorders.

The reviewed RCTs in the present studies revealed several study design strengths. For example, with over 300 participants on average, most examined studies were well powered to detect small to medium effect sizes. Sample sizes were, in most studies, well justified using a priori power analyses. While participant recruitment was mostly via convenient sampling, most studies used random assignment to groups as opposed to cluster random sampling. It is also worth noting that top scoring articles recruited from primary care^19,31^ or clinical settings.^20^ Recruiting from a place of sufficient patient flow rather than a less centralized approach (e.g., posters, online ads) appears an important study design characteristic. One consideration for future improvement may be the inclusion of underrepresented or minoritized groups, as only 3 studies^36,41,42^ focused on a lower economic status population. Data from diverse samples may provide a more externally valid picture of the effectiveness of HWC.

The review of the studies also included areas for study design improvements. For example, strategies to blind researchers and participants for allocation or measurement purposes were rarely applied in these studies. An exception was Ball et al.,^20^ who had a biostatistician perform randomization to groups and only revealed this information to intervention providers, but not to the participants or research team. Appel and colleagues^19^ also blinded assessors to condition by training these staff without allocation knowledge to perform necessary measurements. While a HWC study can never be fully blinded (i.e., coaches and participants inherently know they are involved in treatment), it is valuable to apply available blinding strategies to limit potential for bias towards intervention effectiveness. For most included studies, statistical analyses were well justified and often controlled for important covariates. Intent-to-treat analysis was performed in many of these RCTs. These studies were conducted in several countries (e.g., US, Canada, Denmark, Turkey), enhancing cross-cultural generalizability of findings. In summary, many of these RCTs applied well-conceived study design characteristics.

In terms of intervention design, the reviewed studies generally scored well on number and duration of HWC sessions and program length. This finding is undoubtedly related to our requirement of a minimal program length (i.e., 4 months) and number of sessions for inclusion consideration. These variables provided good potential for a successful coaching process while allowing development of a coach-patient relationship.^3,4^ Adherence to a health coaching strategy and monitoring of health coaching integrity/fidelity were questions receiving moderate rubric scores. Monitoring HWC integrity involves assessing coaching quality (e.g., use of open-ended questions, active listening skills, reflection techniques) and use of specific behavioral change techniques (e.g., motivational interviewing, cognitive behavioral therapy).^4,31,51^ Only 4/18 (22.2%) obesity studies and 2/11 (18.2%) T2D studies reported assessing coaching intervention fidelity which is best done by recording and rating coaching sessions. This process was occasionally done^20,39^ with Nashita et al.^38^ transcribing randomly identified sessions and having coach quality rating of sessions by three independent researchers. Wolever et al.^43^ also described and performed a form of HWC intervention check. Fidelity evaluation of motivational interviewing,^52^ applied in several MI-focused HWC studies,^20,31,53^ is an excellent example of assessing intervention integrity. For example, Sohl and colleagues^51^ recently developed the Health Coaching Index (HCI), an observational tool to assess health coaching fidelity, which may assist in designing and monitoring more effective interventions.

The selected literature, describing HWC intervention effects for obesity and T2D, presents largely positive clinical results. The evaluation of these RCTs, using the HWC-RDR, yielded moderate scores for both theoretical frameworks as well as for overall score. These findings help us to better understand this HWC research, making it possible to suggest these methodological strategies for adoption consideration by future HWC studies:

1. Fully and carefully describe coach characteristics (e.g., training, education, experience, certification) and the HWC intervention (e.g., behavior change strategies).
2. Assess HWC intervention fidelity, using intervention checks and established tools (e.g., HCI),^50^ to ascertain proper delivery of the service
3. Isolate the HWC intervention using controls whenever possible. When HWC is programmatically combined with other lifestyle interventions (e.g., planned dietary change or exercise), the pure effect of HWC becomes difficult to understand.
4. Use allocation concealment and assessor blinding as these methodological techniques are often easy to apply.
5. Use intent-to-treat analysis to allow a fair and unbiased reporting of HWC treatment results. Data can be presented both as “completers” and intent-to-treat analyses.

Application of these research design recommendations should improve the consistency and quality of future HWC research. Accordingly, the HWC-RDR can be useful to not only assess existing research, but also for informing the design of future study. Ideally, the HWC-RDR will evolve and become a better tool for these purposes. The hope is an expanding, high-quality literature base will better define the scope and best practices required to optimize effectiveness of HWC intervention for obesity, T2D, and other lifestyle-related disorders.

### Limitations

The present work presented a novel rubric, which may benefit from further testing. While we assessed the reliability of the rubric through inter-rater scoring and expert input, efforts to provide a higher level of validation should be made with future use. Additionally, we chose to limit our analysis to obesity and T2D studies. This may have jeopardized the generalizability of the present findings as it is also possible studies of other patient populations (e.g., depression or cancer) may score differently using the HWC-RDR. Future research in those patient populations may reveal more generalizable information and should be the target of future research. Scoring bias may also have been introduced by limiting the included studies to RCTs. Other research designs (e.g., case series or pre-post cohort studies) may provide important information about the effectiveness of HWC interventions. However, rubric scores may be different for non-RCT designs and we did not examine these studies. In summary, the HWC-RDR should be a useful tool that will benefit from additional validation and more widespread application. We invite others to test the HWC-RDR and make modifications if needed. For example, some may argue that the concept of coaching fidelity is only marginally captured in the scale (e.g., by the item “Supervision/Fidelity”) because not all components of fidelity may be covered (e.g., autonomy, agency).^51^ Future work in this area may be warranted. Nonetheless, it is our hope that the systematic examination of published HWC research in the present study and the HWC-RDR will be provide practical implications to HWC practitioners and researchers.

## Conclusions

The primary objective of this study was to assess HWC research done with obese and T2D patients. Rather than simply making general comments, we sought to develop a tool to bring consistency and objectivity to assessing HWC. The HWC-RDR is a 28-item rubric permitting systematic evaluation of HWC research strengths and weaknesses within two theoretical frameworks (i.e., study design and intervention design). Other clinical fields have applied similar evaluative processes to their evidence-base.^9-11^ Assessment of literature can be valuable to future research, particularly with an emerging field such as HWC.
